# Relationship Between Parent Vowel Hyperarticulation in Infant-Directed Speech and Infant Phonetic Complexity on the Level of Conversational Turns

**DOI:** 10.3389/fpsyg.2021.688242

**Published:** 2021-08-04

**Authors:** Ulrika Marklund, Ellen Marklund, Lisa Gustavsson

**Affiliations:** ^1^Division of Sensory Organs and Communication, Department of Biomedical and Clinical Sciences, Linköping University, Linköping, Sweden; ^2^Department of Neurology, Speech and Language Clinic, Danderyd Hospital, Stockholm, Sweden; ^3^Division of Speech and Language Pathology, Department of Clinical Science, Intervention and Technology, Karolinska Institutet, Stockholm, Sweden; ^4^Phonetics Laboratory, Stockholm Babylab, Department of Linguistics, Stockholm University, Stockholm, Sweden

**Keywords:** turn-taking, infant-directed speech, phonetic complexity, vowel hyperarticulation, conversational turns, vhh-index, Word Complexity Measure for Swedish, WCM-SE

## Abstract

When speaking to infants, parents typically use infant-directed speech, a speech register that in several aspects differs from that directed to adults. Vowel hyperarticulation, that is, extreme articulation of vowels, is one characteristic sometimes found in infant-directed speech, and it has been suggested that there exists a relationship between how much vowel hyperarticulation parents use when speaking to their infant and infant language development. In this study, the relationship between parent vowel hyperarticulation and phonetic complexity of infant vocalizations is investigated. Previous research has shown that on the level of subject means, a positive correlational relationship exists. However, the previous findings do not provide information about the directionality of that relationship. In this study the relationship is investigated on a conversational turn level, which makes it possible to draw conclusions on whether the behavior of the infant is impacting the parent, the behavior of the parent is impacting the infant, or both. Parent vowel hyperarticulation was quantified using the vhh-index, a measure that allows vowel hyperarticulation to be estimated for individual vowel tokens. Phonetic complexity of infant vocalizations was calculated using the Word Complexity Measure for Swedish. Findings were unexpected in that a negative relationship was found between parent vowel hyperarticulation and phonetic complexity of the immediately following infant vocalization. Directionality was suggested by the fact that no such relationship was found between infant phonetic complexity and vowel hyperarticulation of the immediately following parent utterance. A potential explanation for these results is that high degrees of vowel hyperarticulation either provide, or co-occur with, large amounts of phonetic and/or linguistic information, which may occupy processing resources to an extent that affects production of the next vocalization.

## Introduction

This study investigates the relationship between parents’ infant-directed speech (IDS) and the developing speech production of the infant. In terms of IDS, the focus lies on the specific characteristic of vowel hyperarticulation (VH) often—but not always—found in IDS (e.g., Kuhl et al., 1997; Englund and Behne, 2006; Benders, 2013; Kalashnikova and Burnham, 2018). VH in IDS has been linked to both various language outcomes (Liu et al., 2003; Hartman et al., 2017; Kalashnikova and Burnham, 2018; García-Sierra et al., 2021; Marklund et al., accepted) and immediate facilitation of word recognition (Song et al., 2010), suggesting that it may have an impact on infant language learning and/or processing. On the other hand, from a phonetic point of view, it is to be expected that the degree of VH in parents’ speech varies with the perceptual requirements of the child (Lindblom, 1990; Buz et al., 2016; Marklund and Gustavsson, 2020), whether that be different stages of language development or dynamic in-the-moment fluctuations in focus and attention. This means that VH is a characteristic of IDS which is likely highly susceptible to contextual influence, and that it may well be the case that the varying degrees of VH in parent IDS is an adaptive response to perceived cues from the infant. Therefore, the present study focuses on the directionality of any potential relationship between VH and infant speech production. The specific aspect of infant speech production under investigation is phonetic complexity (PC) of infant vocalizations. The reason for this focus is that a positive correlational relationship between VH of parent IDS and PC of infant vocalizations has previously been established on a subject level (Marklund et al., accepted). This study aims to determine whether such a relationship can be found on the level of individual conversational turns, and if so, if any directionality can be established.

### Infant Behavior Influences Parent IDS

It has been demonstrated that at least some aspects of IDS are part of a feedback loop between parent and infant, in which parents respond to infants’ in-the-moment reactions to their speech by amplifying or attenuating certain IDS characteristics. The pitch of mothers’ IDS to their four-month-old infants can be manipulated by interrupting this feedback loop (Smith and Trainor, 2008). In the study, mothers interacted with their infants *via* monitors. Mothers could both see and hear their infants, while the infants could neither see nor hear their mothers. Instead, research assistants interacted with the infants (out of sight of the mothers) and modulated their interaction based on the momentary pitch characteristics of the mothers’ speech. Providing positive interaction to the infant contingent upon mothers’ high-pitched utterances resulted in more high-pitched utterances from the mother than providing positive interaction to the infant contingent upon mothers’ low-pitched utterances. This suggests that mothers’ pitch modulations in IDS are at least partially a response to the infants’ behavior in response to them (Smith and Trainor, 2008). Similarly, several characteristics of IDS were shown to be attenuated in mothers’ speech when the immediate feedback loop with their two- to four-month-old infants was interrupted by playing previously recorded video instead of live video (Braarud and Stormark, 2008).

It has also been reported that mothers respond differently to different types of infant vocalizations; for example, more mature infant vocalizations elicit a vocal response from the mother more frequently than less mature infant vocalizations (Albert et al., 2018).

When it comes to VH, mothers’ articulation of vowels was impacted when the feedback loop was interrupted as they interacted with their six- to seven-month-old infants (Lam and Kitamura, 2012). Interacting with their infants *via* a video link, mothers showed less VH when their infants were able to see them but not hear them, compared to when the infants could both see and hear them (Lam and Kitamura, 2012).

To summarize, infant behavior—including vocalizations—influences the specific realization of parent IDS in the moment. This has been shown for a number of IDS characteristics, including VH. VH is a result of spontaneous communicative adaptation to the perceptual and linguistic demands of the interlocutor (Lindblom, 1990), in this case the infant. One source of information to the level of infants’ linguistic proficiency is the maturity of their vocalization. Therefore, it is reasonable to posit that PC of infant vocalizations may impact VH in parents’ responses.

### Parent Behavior Influences Infant Language

The linguistic, prosodic, and articulatory modifications that parents use when speaking IDS to their infants are thought to impact both infant language development in the long term and infant language production and perception in the short term. For example, overall amount of IDS in everyday speech input at seven to eleven months is positively correlated with language outcomes at five years of age (Weisleder and Fernald, 2013), and amount of IDS in a one-on-one setting at 11 and 14 months of age is correlated with productive vocabulary at 24 months (Ramírez-Esparza et al., 2014, 2017a) as well as word production at 33 months (Ramírez-Esparza et al., 2017b). IDS also facilitates in-the-moment aspects of language development such as word learning (Ma et al., 2011; Graf Estes and Hurley, 2013), statistical learning (Bosseler et al., 2016) and word recognition (Singh et al., 2009).

Parent social and vocal behavior has also been shown to influence infant vocal behavior of the child. For example, amount of IDS in a one-on-one setting correlates with amount of infant speech output (Ramírez-Esparza et al., 2014), and the prosodic variations in parent IDS are associated with high levels of infant vocalizations (Dunst et al., 2012; Spinelli et al., 2017). Contingent vocal feedback from parents leads to more mature vocalizations, syllabic rather than vocalic in 3-month-olds (Bloom et al., 1987), and syllabic rather than vocalic and more canonical syllables in 8-month-olds (Goldstein et al., 2003). At 9.5 months of age, infants whose mothers responded to vocalizations with words produced more consonant–vowel syllables, while infants whose mothers responded with a long vowel sound produced more fully voiced vocalizations (Goldstein and Schwade, 2008). This demonstrates that the phonetic content of parent utterances can have an impact on the phonetic realization of infant vocalizations.

When it comes to the specific characteristic of VH in parents’ IDS, it has been shown to predict later vocabulary size (Hartman et al., 2017; Kalashnikova and Burnham, 2018), facilitate word recognition (Song et al., 2010), and correlate with concurrent perceptive phonological development (Liu et al., 2003; García-Sierra et al., 2021). However, only one study has so far demonstrated a correlation between VH and infant vocal production, specifically PC of infant vocalizations, at 12 months of age (Marklund et al., accepted).

To summarize, parent behavior—both in terms of IDS realization and temporally contingent social feedback—influences infant language, either long term and/or in the moment. When it comes to VH in parent IDS and PC of infant vocalizations, a positive correlational relationship between them has been shown (Marklund et al., accepted), but any potential momentary impact is yet to be established.

### This Study

This study focuses on the relationship between parent VH and PC of infant vocalizations. A positive relationship between the two has previously been established on a subject level (Marklund et al., accepted), leaving unanswered, and highlighting, the question of directionality. Does the phonetic maturity of infant vocalization influence the articulatory behavior of the parent, and/or does the clarity of parents’ articulation influence the vocal behavior of the infant? Based on previous findings reviewed above, both explanations are plausible. Attempting to shed light on this issue, the present study focuses on the relationship between parent VH and infant PC on a turn level. The VH of parent utterances immediately preceding and following infant vocalizations is calculated and related to the PC of the vocalization.

This study uses vhh-index, a measure of VH that normalizes across vowel type and speaker, and thus makes it possible to estimate and compare VH of individual vowel tokens. This measure has been used in a previous study on VH in Swedish IDS to 12-month-olds, where it was motivated from phonetic theory and compared to traditional measures of VH for validation purposes (Marklund and Gustavsson, 2020). The rationale for using the vhh-index in the current study is that, contrary to traditional measures, it is calculated on the level of individual vowel tokens, permitting analysis on a turn level. Previous studies on the relationship between VH and infant language have used vowel space area (Liu et al., 2003; Hartman et al., 2017; Kalashnikova and Burnham, 2018; García-Sierra et al., 2021; Marklund et al., accepted). Vowel space area is calculated on a subject level and can thus not be analyzed on the level of individual turns. On a subject level, both vhh-index calculated on all vowel types and vhh-index calculated only on point vowels have previously shown comparable results to vowel space area measures of VH (Marklund and Gustavsson, 2020).

The measure of infant vocalization maturity used in the present study is the Word Complexity Measure for Swedish (WCM-SE; Marklund et al., 2018). The WCM-SE can be used as a measure of phonological maturity, that is, it may be used to measure the stability of a developing phonological system. However, since the infants taking part in the present study are only 12 months of age and as such are not expected to have much of a phonological system in place yet, the WCM-SE is in this case used to estimate PC of infant vocalizations. This is possible since the phonological complexity parameters included in the WCM-SE are also based on PC as detailed in a previous paper (Marklund et al., 2018).

## Materials and Methods

### Participants

Nineteen infants and their parents participated in this study (9 girls, 10 boys; 12 mothers, 7 fathers). At the time of recording the material, the infants were approximately 12 months old (mean = 12.0, range = 11.5–12.3, SD = 0.2). All infants were born full term (within three weeks of due date) and monolingual (defined as both parents speaking only Swedish with the infant). The majority of the parents (*n* = 15) had university education, and all had completed high school (which entails three non-obligatory years of education after the mandatory nine to ten years of basic education in Sweden). The participants constitute a subset of a larger group of subjects (*n* = 72), taking part in a longitudinal study in which parent–child dyads were recorded during free play every three to six months, when the child was between three months and three years.^1^

Participants were selected for inclusion in the present study if (a) there was a recording from the 12-month visit, (b) the infant was monolingual, and (c) there was sufficient ADS material (recorded at the 27-month visit, from the same parent as in the 12-month visit) to include in the VH analysis. The study has been approved by the Regional Ethics Review Board (2015/63-31). For the original longitudinal study, recruitment was conducted *via* mail. Addresses of infants in the appropriate age range and living in the greater Stockholm area were obtained *via* the Swedish Tax Agency, and their parents were invited to participate in the study. Parents received memory sticks with all their audio and video recordings as thanks for their participation in the longitudinal study.

### Recordings

Audio and video recordings of parent–infant interaction were made at Stockholm Babylab, the Phonetics Laboratory, Stockholm University. Parent–infant dyads (one parent and the infant) were recorded in a comfortable carpeted studio equipped with age-appropriate furniture and toys. Video and audio recordings were made with three cameras (Canon XA10) mounted on the walls of the studio to capture all angles of the parent interacting with the infant. A fourth camera (GoPro Hero3), attached to the parent’s chest, enabled video uptake of the infant facing the parent. To capture high-quality audio, an additional three microphones were used. Omnidirectional wireless lavalier microphones (Sennheiser EW 100 G2) were mounted on parent and infant, and one room microphone (AKG SE 300 B) was mounted on a high shelf. In the present study, audio from the two lavalier microphones was used, since this enables high-quality close-up recordings of the parent’s speech and the infant’s vocalizations with minimal interference from the other speaker.

Each infant was recorded together with the parent for approximately 10 min, providing the infant vocalizations and the parent IDS material for the current study. The experimenter instructed parents to interact, play, and talk with their infant as they typically would at home. After instructions and equipment arrangements, the experimenters left the studio, closed the door, and monitored the session from the adjacent control room.

### Vowel Hypo- and Hyperarticulation Estimations in Parent Speech Material

Estimation of VH in parent’s IDS was performed as a part of a previous study (Marklund and Gustavsson, 2020), and detailed information about the procedure can be found there. In brief, parent speech was quasi-orthographically transcribed by a team of researchers and research assistants, then automatically segmented, converted to IPA, and aligned with their audio files using the web service WebMAUS General 5.33 of the Bavarian Archive for Speech Signals at the University of Munich (Schiel, 1999; Kisler et al., 2017). Formants were estimated for the audio recordings, using default settings in Praat (Boersma and Weenink, 2018), except for formant ceiling and number of expected formants which were varied as part of a procedure for more robust formant estimation *via* formant ceiling optimization (Escudero et al., 2009). Since reliability of formant estimations decreases considerably with higher fundamental frequency (*f*_o_), vowel tokens with a median *f*_o_ exceeding 350 Hz were excluded (Monsen and Engebretson, 1983).

VH was quantified using a novel measure, the vhh-index, which entails speaker and vowel normalization, so that VH can be estimated for each individual vowel token (Marklund and Gustavsson, 2020). A midpoint of the acoustic vowel space defined by *F*_1_ and *F*_2_ was calculated for each individual participant based on all available tokens (in this case vowels found in both ADS and IDS). This point in space served as the absolute zero point on a scale of VH, representing extreme hypoarticulation. For each vowel type, the mean formant values were then calculated, and that point in acoustic space served as the midpoint of an individual VH scale for each vowel type. The zero point and the midpoint were used to calculate a theoretical maximum of the individual VH scale, representing hyperarticulation. Individual vowel tokens were then placed along this scale and given a vhh-index based on where they appear in the acoustic space.

### Phonetic Complexity Estimations in Infant Speech Material

Infant vocalizations were transcribed in ELAN 5.8-5.9 (Sloetjes and Wittenburg, 2008) using IPA. The transcriptions were performed by two experienced phoneticians (authors UM and LG) according to a protocol developed for compatibility with WCM-SE (Marklund et al., 2018).

**Figure 1:**
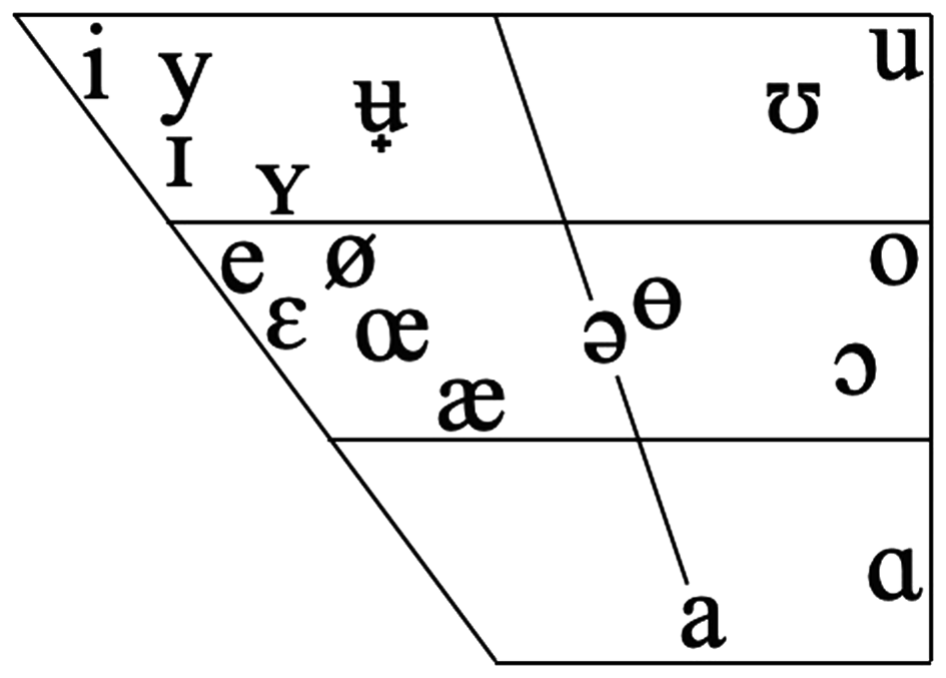
The Swedish vowels used in the transcription. Vowels not recognizable as any of those phonemes were marked as “V.” Adapted from IPA Chart from International Phonetic Association.

The protocol entailed transcribing all sounds present in the Swedish phoneme inventory as described in Engstrand (1999), with the addition of a number of other common allophones (Table 1 and Figure 1). The Swedish vowel inventory consists of seventeen vowel qualities, some of which are considered pairs of phonemically contrasted long and short vowels. However, this phonematic quantity distinction is not only based on the duration of the vowel relative to adjacent consonants, but also its spectral quality (see Elert, 1964; Hadding-Koch and Abramson, 1964). Only the spectral quality was transcribed, and no length signs were used, since vowel quality is sufficient to distinguish the vowels that are awarded points in WCM-SE (Figure 1). Segments that were not possible to interpret as Swedish phonemes were annotated with “C” if consonant-like and “V” if vowel-like. If it was not possible to determine whether the sound was a consonant or a vowel, it was denoted with a square. Syllable boundaries and primary stress were also marked up in each vocalization.

**Table 1:** The Swedish consonants used in the transcription. Consonants not recognizable as any of those phonemes were marked as “C.” Adapted from IPA Chart from International Phonetic Association.

	Bilabial		Labiodental		Dental		Retroflex		Alveolar		Palatal^†^		Velar		Uvular		Glottal	
Plosive	p	b			t	d	ʈ	ɖ					k	ɡ				
Nasal		m				n		ɳ						ŋ				
Trill						r										ʀ		
Tap/flap						ɾ												
Fricative			f	v	s		ʂ	ʐ			ʝ					ʁ	h
Approximant									ɹ								
Lat. approximant						l		ɭ										

† *also /ɧ/ (voiceless dorso-palatal/velar fricative) and /ɕ/ (voiceless alveolo-palatal fricative)*.

All infant vocalizations were transcribed. They could consist of words, syllables, babbling, or isolated speech sounds. Laughter, crying, fuzzing, coughing, effort sounds, and vegetative sounds such as breathing, sneezes, and hiccups were not transcribed. Overlapping speech and distorted sounds were excluded. Boundaries between vocalizations were based on silence (pause or breath) and thus not dependent on interpretation of lexical content or on other linguistic information such as intonation.

Two recordings were annotated by both annotators independently to check inter-transcriber agreement. Percentage of matching characters for each transcribed vocalization was compared. Characters were IPA consonants, IPA vowels (treated as a single category in the inter-rater comparison, unless they were long, front, and rounded, i.e., relevant to the WCM-SE measure, in which case their vowel quality was taken into account), syllable markers, stress markers, and vocalization boundary markers. Inter-transcriber agreement of which vocalizations were transcribed was 70%, and out of those the average transcription inter-transcriber agreement was 78%.

To operationalize complexity in infant vocalizations, the WCM-SE was used (Marklund et al., 2018). Based on a number of phonological/phonetic complexity parameters in three domains, a complexity score is calculated for each vocalization based on how many of the parameters are present in it (Table 2). For example, the Swedish word *elefant* (“elephant”) produced as ele'fant results in 6 WCM-SE points, since it has more than two syllables, non-initial stress, a word final consonant, 1 consonant cluster, 1 liquid, and 1 fricative. *Sko* (“shoe”) produced as sku: results in 3 points and màma (“mommy”) produced as màma results in 0 points. WCM-SE points were calculated for each vocalization based on the transcriptions using a script written in R 3.5.0-4.0.2 (R Core Team, 2018). Examples of WCM-SE calculations of the material in this study can be seen in Table 3.

**Table 2:** The WCM-SE measure as implemented in the present study, based on Marklund et al. (2018). Each transcribed vocalization was given a WCM-SE score, after which a subject mean score was calculated.

Domains	Complexity parameter	N points
Word patterns	>2 syllables	1 per vocalization
	Non-initial stress	1 per vocalization
Syllable structures	Word-final consonant	1 per vocalization
	Consonant cluster^†^	1 per occurrence
Sound classes	Velar consonant [k], [ɡ], [ŋ], [ɧ]	1 per occurrence
	Liquid [l], [ɭ], [ɹ]	1 per occurrence
	Fricative^‡^ [f], [v], [s], [ʐ], [ʁ], [ʂ], [ʝ], [h], [ɧ], [ɕ]	1 per occurrence
	Voiced fricative [v], [ʐ], [ʁ], [ʝ]	1 per occurrence
	Trill [r], [ʀ]	3 per occurrence
	Long, front, rounded vowel [y], [ø], [ʉ̟]	1 per occurrence

† *Only consonant clusters within syllables were counted*.

‡ *Vocalization-final [h] excluded, since that is likely to be release of breath*.

**Table 3:** Examples of transcriptions of infant vocalizations, and WCM-SE calculations for the vocalizations. Syllable onsets are denoted by “.” and stress by “ˈ”.

Example transcription	Complexity parameters	Points
.ə.ˈbm	Non-initial stress, word-final consonant, consonant cluster	3
.ˈC.C	Word-final consonant	1
.ˈtɛ.kɛ.tæ	>2 syllables, velar consonant [k]	2
.ˈhɪ.Cɪ.V	>2 syllables, fricative [h]	2
.hɔŋ.ɡɛ.ˈjɛ	>2 syllables, non-initial stress, velar consonants [ŋ] and [ɡ], fricative [h]	5
ˈV.V	–	0

### Data and vhh-index Measures

Data consist of infant vocalization WCM-SE score and vhh-index measures of the preceding and following parent utterances. Cases where an infant vocalization was preceded or followed by another infant vocalization were excluded. Since the vhh-index is novel, and token-based measures of VH have not been used previously, a number of vhh-index measures were included for exploratory purposes. All VH measures were calculated on the level of utterances, that is, they are based on all vowels for which vhh-index could be calculated within a single parent utterance. The measures were mean vhh-index, max vhh-index, vhh-index range, hyperarticulation ratio (number of vowels with vhh-index > 50 over total number of vowels), weighted mean vhh-index, and weighted max vhh-index. The weighted mean and max vhh-index entails multiplying the vhh-index with the duration of the vowel, to give more weight to longer vowels and less weight to shorter vowels. The purpose of weighting the vowel tokens like this is to reflect their relative salience in the speech signal; a vowel with long duration entails longer exposure to its particular spectral properties than a vowel with shorter duration.

### Analyses

The analyses were performed using linear mixed models. Linear mixed models are conceptually similar to regular linear regression models, except that they also account for within-subject variation, essentially allowing the model to disregard between-subject variation in favor of variation related to the independent variable.

Two linear mixed effects regressions were calculated for each of the measures of vhh-index on utterance level (mean, max, range, ratio, weighted mean, and weighted max), one on data points in which the parent utterance preceded the infant vocalization (parent–infant turns), and one on data points in which the parent utterance followed the infant vocalization (infant–parent turns). In the case of parent–infant turns, the predicted variable was infant vocalization WCM-SE score, and the fixed effects variable was the parent utterance vhh-index measure. In the case of infant–parent turns, the predicted variable was the parent utterance vhh-index measure, and the fixed effects variable was infant vocalization WCM-SE score. In both cases, random variable was participant, that is, parent–infant dyad (intercept only).

## Results

Data points with infant vocalizations that were outliers (thresholds: Q ± 3^*^IQR) in terms of WCM-SE score were removed (*n* = 4), leaving 580 unique vocalizations to be included in the analysis, with a mean WCM-SE score of 1.6. Outliers in terms of formant values were removed on a vowel token level prior to calculating the vhh-index, as were tokens with *f*_o_ exceeding 350 Hz (Marklund and Gustavsson, 2020). The number of vowel token outliers removed was 886, and the number of high *f*_o_ tokens removed was 580, leaving a total of 7,688 vowel tokens. The average vhh-index for parent utterances was thus calculated on the remaining tokens within each utterance, and as such no utterances were considered outliers. The number of unique parent utterances included in the analysis was 855, and the average vhh-index was 73.7. In the analysis, 379 parent–infant turns and 476 infant–parent turns were included.

For mean vhh-index, max vhh-index, vhh-index range, and vhh-index ratio, no significant results were found (Tables 4–7). The weighted mean vhh-index of the parent utterance significantly predicted the infant vocalization WCM-SE score in parent–infant turns (Table 8). Surprisingly, the relationship was negative, with a change of −0.022 (95% CI -0.041| -0.003) in WCM-SE score for every increase of 1 in weighted mean vhh-index. This means that a difference between neutral articulation (vhh-index = 50) and hyperarticulation (vhh-index = 100) is associated with a difference of 1.1 WCM-SE points in the infant vocalization, reflecting the addition of one complex element. No significant relationship was found between parent utterance weighted mean vhh-index and infant vocalization WCM-SE score in infant–parent turns (Table 9). The same patterns were found for weighted max vhh-index (Table 5), that is, a change of −0.012 (95% CI -0.024| -0.001) in WCM-SE score for every increase of 1 in weighted max vhh-index in parent–infant turns, but no significant relationship was found in infant–parent turns.

**Table 4:** Summary of the fixed effects of the analysis of the measure mean vhh-index in parent–infant turns **(A)** and infant–parent turns **(B)**. No significant effects were found.

	Est.	SE	*t*
**A. Fixed effects: Parent–infant turns**
Intercept	1.36	0.25	5.55
Parent utterance mean vhh-index	<−0.01	< 0.01	−0.27
**B. Fixed effects: Infant–parent turns**			
Intercept	72.8	3.37	21.6
Infant vocalization WCM-SE score	0.41	1.18	0.34

**Table 5:** Summary of the fixed effects of the analysis of the measure max vhh-index in parent–infant turns **(A)** and infant–parent turns **(B)**. No significant effects were found.

	Est.	SE	*t*
**A. Fixed effects: Parent–infant turns**
Intercept	1.29	0.24	5.47
Parent utterance max vhh-index	<0.01	<0.01	0.38
**B. Fixed effects: Infant–parent turns**			
Intercept	106.8	6.75	15.8
Infant vocalization WCM-SE score	1.66	2.07	0.80

**Table 6:** Summary of the fixed effects of the analysis of the measure vhh-index range in parent–infant turns **(A)** and infant–parent turns **(B)**. No significant effects were found.

	Est.	SE	*t*
**A. Fixed effects: Parent–infant turns**
Intercept	1.28	0.22	5.82
Parent utterance vhh-index range	<0.01	<0.01	0.93
**B. Fixed effects: Infant–parent turns**			
Intercept	61.4	6.15	9.98
Infant vocalization WCM-SE score	1.35	2.13	0.64

**Table 7:** Summary of the fixed effects of the analysis of the measure vhh-index ratio in parent–infant turns **(A)** and infant–parent turns **(B)**. No significant effects were found.

	Est.	SE	*t*
**A. Fixed effects: Parent–infant turns**
Intercept	1.35	0.26	5.28
Parent utterance vhh-index ratio	−0.03	0.21	−0.16
**B. Fixed effects: Infant–parent turns**			
Intercept	0.68	0.02	31.0
Infant vocalization WCM-SE score	<−0.01	0.01	−0.86

**Table 8:** Summary of the fixed effects of the analysis of the measure weighted mean vhh-index in parent–infant turns **(A)** and infant–parent turns **(B)**. According to the t-as-z approach to estimate statistical significance (thresholds ±1.96; Luke, 2017), the effect of weighted mean vhh-index in the parent utterance can be considered significant in infant–parent turns (marked by an asterisk).

	Est.	SE	*t*
**A. Fixed effects: Parent–infant turns**
Intercept	1.49	0.22	6.64
Parent utterance weighted mean vhh-index	−0.02	0.01	−2.24*
**B. Fixed effects: Infant–parent turns**			
Intercept	9.13	0.65	14.0
Infant vocalization WCM-SE score	−0.31	0.30	−1.04

**Table 9:** Summary of the fixed effects of the analysis of the measure weighted max vhh-index in parent–infant turns **(A)** and infant–parent turns **(B)**. According to the t-as-z approach to estimate statistical significance (thresholds ±1.96; Luke, 2017), the effect of weighted max vhh-index in the parent utterance can be considered significant in infant–parent turns (marked by an asterisk).

	Est.	SE	*t*
**A. Fixed effects: Parent–infant turns**
Intercept	1.49	0.23	6.49
Parent utterance weighed max vhh-index	−0.01	0.01	−2.06*
**B. Fixed effects: Infant–parent turns**			
Intercept	15.3	1.22	12.6
Infant vocalization WCM-SE score	−0.13	0.52	−0.26

## Discussion

The results show a negative relationship between parent VH in IDS to their 12-month-old infants and the PC of infant vocalizations on a turn level; specifically, the more hyperarticulated a parent utterance is, in terms of mean and max vhh-index weighted for vowel duration, the less phonetically complex the following infant vocalization is, in terms of WCM-SE score.

This is a somewhat surprising finding since previous findings on the same data show a positive relationship between parent VH and PC of infant vocalizations on the level of individual dyads (Marklund et al., accepted). Based on previous findings, it was expected that if any relationship was found, it would be a positive one, that is, a high degree of VH in the parent utterance would be followed by high PC in the infant vocalization, or high PC in the infant vocalization would be followed by a high degree of VH in the parent utterance.

In the previous study, the positive correlation between infants’ WCM-SE scores and parents’ VH (measured in vowel space area) could indicate that parents’ articulation impact infants’ production and/or that infants’ production impact parents’ articulation, or that a third, underlying variable mediates the relationship. For example, it is possible that articulatory adaptiveness is a specific realization of a general communicative adaptiveness, and that other components of this general adaptiveness may be the driving factors for any potential benefit for language development, rather than VH in itself.

In the present study, both the direct impact of VH on a turn level and the directionality of any potential effect were investigated. The negative relationship that was found between infant WCM-SE score and parent vhh-index suggests that there is a direct, in-the-moment causality between the two, and directionality of the effect was indicated by the fact that the effect was only significant in parent–infant turns.

Had the effect been significant in both directions, one potential interpretation could have been that parents are responsive and use a high degree of VH to support the linguistic needs of infants with less mature vocalizations overall. However, previous studies have shown that parent VH is typically attenuated rather than increased in interaction with atypically developing infants or infants at risk for developmental delays (Lam and Kitamura, 2010; Kalashnikova et al., 2018). It is therefore not necessarily the case that increased VH would be expected in response to immature vocalizations either. Regardless, the effect was found only in parent–infant turns, suggesting that it is the parents’ articulation that has an impact on the infant vocalization.

There is no reason to believe that an infant would try less hard in their production as a direct response to high degrees of VH in the preceding parent utterance. However, high levels of hyperarticulation in the input might mean more or novel phonetic information to process for the infant. This could potentially leave less energy or focus for the infant in regard to the next task, that is, production of the next vocalization. This is in line with the *resource limitation hypothesis* which states that task demands (in addition to the developmental level of the infant) may impact attention to critical details of the speech signal (Curtin et al., 2011). Another possibility is that there might be other characteristics of parent utterances with high VH that elicit other types of responses from the infant. As an example, also in line with the resource limitation hypothesis, VH typically occurs when introducing words that are rare or have dense phonological neighborhoods (Munson and Solomon, 2004), and so utterances with incidental high VH may claim additional processing resources because they introduce new or complex linguistic information.

There are limitations to this study that should be acknowledged. The study has a relatively small sample size, although in line with previous similar studies (e.g., Liu et al., 2003; Hartman et al., 2017; Kalashnikova and Burnham, 2018; García-Sierra et al., 2021). However, by using the vhh-index as the measure of VH and linear mixed models for the analysis, multiple data points per participant could be used. Nevertheless, the small sample may be a contributing factor to the null findings in most of the vhh-measures.

There are a few things to take into consideration with regards to the complexity measure of the infant vocalizations, the WCM-SE score (Marklund et al., 2018). The choice to use this measure, even if the infants that took part in the study were too young to have a phonological system in place, was motivated because the measure is based on phonetic principles. Nevertheless, it makes assumptions of an underlying phonological system in the making and this may affect the complexity score. An additional issue is that transcription of young infants’ vocalizations is notoriously difficult. The choice here was to use quite broad phonetic transcriptions compatible with the WCM-SE grading, but phonetic segments are quite detailed and perhaps not optimal representations of early vocalizations. On a related note, the interrater reliability in this study can be considered low, especially if compared to transcriptions of adult speech. It can, however, be considered a reasonable level of agreement when transcribing young infants’ vocalizations on this level of detail. Previous studies with less detailed annotations (e.g., canonical babbling vs. non-canonical babbling or syllable counts) report between 70 and 84% interrater reliability (Warlaumont and Ramsdell-Hudock, 2016; Lieberman et al., 2019), and the agreement in this study lies within that range (70 and 78%).

In addition, the study uses a method to quantify VH in the parent speech, the vhh-index, which has only recently been developed (Marklund and Gustavsson, 2020) and not yet evaluated thoroughly. There are ways in which this measure could be improved on theoretical grounds, for example, the current algorithm for placing tokens in the hyperarticulation direction of the hypo-/hyper-scale does not punish deviations in one of the two formant frequencies, and such deviations should in theory impact the calculated vhh-index. Applying this novel measure in multiple ways increases the risk for spurious significant findings, but since no previous research exists as basis for methodological choices, this was deemed justified in this study.

Furthermore, high *f*_o_ is one of the most consistently reported characteristics of IDS (e.g., Fernald et al., 1989); however, tokens with *f*_o_ of more than 350 Hz were excluded since they are highly unreliable in terms of formant measures (Monsen and Engebretson, 1983). These exclusions introduce a potential validity issue. However, keeping high *f*_o_ tokens in the analysis is not a viable option, since the fact that their formant estimation likely are inaccurate would be a major reliability issue.

There is also the possibility that the fact that recordings were made in a laboratory impacted the way that parents and infants interacted. However, previous research has shown both that young children speak similarly in different contexts such as laboratory setting or at home (Stevenson et al., 1986; Bornstein et al., 2000), and that mothers’ speech is similar in laboratory setting and home, in particular after the first few minutes (Stevenson et al., 1986; although see Belsky, 1980 for differences in amount of parent speech in laboratory vs. home settings). In this study, parents were familiar with the laboratory setting since the families visited regularly to make recordings as part of the longitudinal study. The recording used in this study was made during parents’ and infants’ third or fourth visit to the laboratory. The literature also confirms that the presence of an observer does not necessarily negatively affect the nature of interaction (Gardner, 2000). Parents in this study were aware that various aspects of parent-child interaction were to be studied but they were not informed specifically about any analyses of articulation, which minimizes the risk that they would articulate in a way that was less natural to them.

The unexpected findings are difficult to interpret and explain in the light of existing knowledge, and one reason is that this study is the first of its kind. Given this, as well as the limitations listed above, it is premature to talk about new insights into the relationship between VH in parent IDS and infant speech production based on the findings this study. They have, however, contributed to new thoughts about how perceptual processing demands potentially impacts infant production, which need to be addressed in future studies, together with further evaluation of the VH and PC measures used in this study.

In conclusion, the present study reports a negative relationship between VH in parent utterances and PC in immediately following infant vocalizations. No relationship was found between PC in infant vocalization and VH of the immediately following parent utterance. That is, a negative relationship between parent VH and infant PC was found on the level of conversational turns, and the directionality suggested was that parent utterances influence infant vocalizations rather than the opposite.

## Data Availability Statement

Tabular data generated for this study are available at the Open Science Framework at: https://osf.io/jw32d/.

## Ethics Statement

The studies involving human participants were reviewed and approved by The Regional Ethics Committee in Stockholm, Sweden (2015/63-31). Written informed consent to participate in this study was provided by the adult participants and the infant participants’ legal guardian/next of kin.

## Author Contributions

All authors contributed to the article and approved the submitted version. UM, EM, and LG: study design, drafting the manuscript, and critical revisions of the manuscript. UM and LG: data collection (part) and transcriptions. EM: data processing and analyses. All authors approved the submission.

## Conflict of Interest

The authors declare that the research was conducted in the absence of any commercial or financial relationships that could be construed as a potential conflict of interest.

## Publisher’s Note

All claims expressed in this article are solely those of the authors and do not necessarily represent those of their affiliated organizations, or those of the publisher, the editors and the reviewers. Any product that may be evaluated in this article, or claim that may be made by its manufacturer, is not guaranteed or endorsed by the publisher.

## Footnotes

^1^The longitudinal study was part of the MINT-project (MAW 2011.0070, PI Gerholm).
